# A Cell-Based Strategy for Bioactivity Determination of Long-Acting Fc-Fusion Recombinant Human Growth Hormone

**DOI:** 10.3390/molecules24071389

**Published:** 2019-04-09

**Authors:** Wenrong Yao, Lei Yu, Wenhong Fan, Xinchang Shi, Lan Liu, Yonghong Li, Xi Qin, Chunming Rao, Junzhi Wang

**Affiliations:** National Institutes for Food and Drug Control, No. 2, Tiantan Xili, Beijing 100050, China; yz1322@126.com (W.Y.); yulei@nifdc.org.cn (L.Y.); fanwh@nifdc.org.cn (W.F.); shixc@nifdc.org.cn (X.S.); liulan@nifdc.org.cn (L.L.); liyh@nifdc.org.cn (Y.L.); qinxi@nifdc.org.cn (X.Q.)

**Keywords:** growth hormone, long-acting Fc-fusion recombinant human growth hormone, method validation, cell-based bioassay, reporter gene assay

## Abstract

The long-acting growth hormone (LAGH) is a promising alternative biopharmaceutical to treat growth hormone (GH) deficiency in children, and it was developed using a variety of technologies by several pharmaceutical companies. Most LAGH preparations, such as Fc fusion protein, are currently undergoing preclinical study and clinical trials. Accurate determination of bioactivity is critical for the efficacy of quality control systems of LAGH. The current in vivo rat weight gain assays used to determine the bioactivity of recombinant human GH (rhGH) in pharmacopoeias are time-consuming, expensive, and imprecise, and there are no recommended bioassays for LAGH bioactivity in pharmacopoeias. Therefore, we developed a cell-based bioassay for bioactivity determination of therapeutic long-acting Fc-fusion recombinant human growth hormone (rhGH-Fc) based on the luciferase reporter gene system, which is involved in the full-length human GH receptor (hGHR) and the SG (SIE and GAS) response element. The established bioassay was comprehensively validated according to the International Council for Harmonization (ICH) Q2 (R1) guidelines and the Chinese Pharmacopoeia, and is highly precise, time-saving, simple, and robust. The validated bioassay could be qualified for bioactivity determination during the research, development, and manufacture of rhGH-Fc, and other LAGH formulations.

## 1. Introduction

Growth hormone (GH), also known as somatotropin, is pituitary-derived anabolic hormone that regulates a number of metabolic processes involved in growth and development [1,2]. The specific responses to body growth and metabolism act via multiple GH signal transduction pathways. Upon binding to the GH receptor, the GH-associated janus kinase 2 (JAK2) tyrosine kinase is activated, and then the signaling proteins to GH receptor (GHR)–JAK2 complexes are recruited by phosphorylated tyrosines and subsequently induce signal transducers and activators of transcription (STAT), mitogen-activated protein kinases (MAPK), and phosphatidylinositol 3 kinase (PI3K) intracellular signaling pathways [3,4]. GH deficiency (GHD) causes growth retardation in children and metabolic dysfunction in adults. Recombinant human GH (rhGH) is a 22 kDa polypeptide that consists of 191 amino acids. Treatment with rhGH in children with GHD has been well established since its approval by the United States Food and Drug Administration (FDA) in 1985 [1,5]. 

rhGH has a short-circulating half-life of only a few hours in the human body, and the therapeutic regimens rely on daily subcutaneous injections that can be burdensome, inconvenient, and painful for some patients. Further, daily injections may cause non-adherence over time and result in decreased therapeutic efficacy [6,7,8]. Therefore, long-acting growth hormone (LAGH) formulations with extended half-lives have been developed to improve adherence and simplify dosing schedules, which were existing disadvantages of rhGH [6,8,9,10,11,12,13]. Regarding the LAGH formulations, several forms have been developed by increasing the effective size of rhGH and reducing its rate of clearance from the body, such as Fc fusion rhGH and PEGylated rhGH. Additionally, Fc domain binding to the neonatal Fc receptor (FcRn) prolongs the serum half-life of rhGH-Fc, and salvages the protein from being degraded in endosomes [7,14,15,16].

Due to its unique pharmacokinetics and pharmacodynamics, and inherent complexity, the functional characterization of LAGH is especially required to closely monitor bioactivity, bioidentity, and other types of physicochemical characterization [6,9,11,16,17]. Nowadays, the bioassay for rhGH bioactivity highly relies on in vivo rat weight gain, as described in the US Pharmacopeia and the Chinese Pharmacopoeia, and it is time-consuming, expensive, and imprecise. Additionally, the in vitro rat Nb2-11 cell-based proliferation assay is recorded in the US Pharmacopeia. The limitation of the proliferation assay is that the lactogenic bioactivity of rhGH, rather than the bioactivity of rhGH, is determined through responsiveness to prolactin receptors expressed on the Nb2-11 surface [18]. In regard to the bioactivity of LAGH, there are no recommended bioassays in the Chinese Pharmacopoeia, although the PEGylated-rhGH (Jintrolong) LAGH was commercially developed and marketed in China [7]. Consequently, the development of an animal-free bioassay with a low coefficient of variation (CV) that is time-saving and simple to carry out needs to be explored to facilitate the determination of bioactivity for LAGH formulations. The unchanged specificity, lower variability, and simpler procedures of reporter gene assays (RGAs) compared with animal assays render them excellent alternatives for the bioactivity determination of biopharmaceuticals [19,20]. Actually, RGAs focused on the hGHR-LHRE reporter system are suitable for the measurement of the biological activity of serum GH, rhGH, and ligand–receptor fusion of GH. However, transient transfection and the lack of method validation have probably impeded the drive to determine the bioactivity of therapeutic rhGH or LAGH [18,21,22,23]. 

Here, we develop a cell-based bioassay to determine the bioactivity of therapeutic rhGH-Fc using RGA and comprehensively validate it according to the International Council for Harmonization (ICH) Q2 (R1) guidelines and the Chinese Pharmacopoeia.

## 2. Materials and Methods

### 2.1. Cells and Materials

The human embryonic kidney 293 (HEK293) cell line (CRL-1573™) was obtained from the American Type Culture Collection (Manassas, VA, USA). RPMI1640, fetal bovine serum (FBS), Geneticin (G418), and hygromycin B were purchased from Gibco (Grand Island, NY, USA). Human growth hormone receptor (hGHR) plasmid was obtained from OriGene Technologies (Beijing, China). ViaFect™ transfection reagent was purchased from Promega (Madison, WI, USA). The Britelite Plus Reporter Gene Assay System was obtained from PerkinElmer (Waltham, MA, USA). The SG-luciferase reporter plasmid (including SIE and GAS response elements) [19], an in-house reference of rhGH-Fc, rhGH-Fc, rhGH, Fc-fusion recombinant human erythropoietin (rhEPO-Fc), Fc-fusion vascular endothelial growth factor receptor (VEGFR-Fc), Fc-fusion recombinant human interleukin 15 (rhIL15-Fc), and Fc fusion recombinant human GLP1 (rhGLP-Fc) were stored at 4 °C or –80 °C in our laboratory. The 96-well flat clear bottom white polystyrene TC-treated microplates were purchased from Corning (New York, NY, USA). 

### 2.2. Preparation of Responsive Cells to rhGH-Fc

The HEK293 cells were cultured in DMEM supplemented with 10% FBS in an incubator atmosphere of 5% CO_2_ at 37 °C, and they were split every 3 days. The SG-luciferase reporter plasmid and hGHR plasmid were transfected simultaneously into HEK293 cells using ViaFect™ transfection reagent according to the manufacturer’s instructions. The cells expressing SG-luciferase and hGHR received regular changes of DMEM-10% FBS with hygromycin B (300 μg/mL) and G418 (1000 μg/mL) and were continuously cultured for at least for 5 weeks. Then, a clonal cell line derived from a single cell was produced by limiting the dilution from the stably transfected HEK293 cells using 0.5 cells per well in a 96-well plate. After isolating the clones, assessments through rhGH-Fc stimulation were performed. The cells, namely HEK293-Luc, were highly responsive to rhGH-Fc. The HEK293-Luc cells were maintained in DMEM-10% FBS with 200 μg/mL of hygromycin B and 500 μg/mL of G418. Meanwhile, the HEK293 cell line only transfected with SG-luciferase plasmid without hGHR plasmid was used as the control group. 

### 2.3. Bioactivity Assay

The bioassay of rhGH-Fc was performed in vitro as a cell-based bioassay as described previously [20]. In brief, 4 × 10 ^4^ cells in 70 μL RPMI1640 were added to each well in a 96-well cell plate, and they were incubated for 18–22 h at 37 °C in a 5% CO_2_ incubator. An rhGH-Fc (30 mg/mL) and in-house rhGH-Fc reference (31.4 mg/mL) were diluted by serial 3-fold dilutions with RPMI1640 from pre-diluted starting concentrations of 30,000 ng/mL. Then, 70 µL of diluted rhGH-Fc was added to cells. It should be noted that the final working concentration of rhGH-Fc was 15,000 ng/mL, and the final concentration ranged from 6.9 ng/ml to 15,000 ng/mL. After 5 h of incubation at 37 °C in a 5% CO_2_ incubator, the supernatants were removed, and 60 µL Britelite Plus Reporter Gene Assay reagent was added to each well. After 5 min of mixing the reagents under subdued light conditions, the relative luciferase units (RLU) were determined by a Luminoscan Ascent plate reader (Molecular Devices, San Jose, CA, USA).

The sigmoidal curves, signal-noise-ratio (SNR), and 50% effective concentration (EC_50_) were calculated through a four-parameter method. The SNR was indicated by the ratio of the RLU of the top asymptote to the bottom asymptote. The relative bioactivity of rhGH-Fc was shown as the ratio of the EC_50_ values of an in-house reference to the EC_50_ values of the rhGH-Fc samples. 

### 2.4. Preparation of Forced Degradation of rhGH-Fc

The forced degradation of rhGH-Fc was applied under thermal stress conditions as described previously [20]. The rhGH-Fc samples were kept at 60 °C for 1, 3, 5, and 7 days to induce degradation. After that, the degraded rhGH-Fc was used to determine the bioactivity according to the procedure given under Section 2.3. 

### 2.5. Data Analysis and Statistics

All the statistical tests were performed using GraphPad Prism 7.0 (GraphPad Software Inc., San Diego, CA, USA). Comparisons between two groups were performed using a two-tailed Mann–Whitney test, and multiple comparisons were performed using a Kruskal–Wallis test with Dunn’s multiple comparisons. *p*-values < 0.05 were deemed to be statistically significant. Each experiment was performed in triplicate. The RLU of each concentration indicates the mean of three replicates. The mean ± SD is shown on each curve.

## 3. Results

### 3.1. Generation of Stable rhGH-Fc-Responsive Cell Line

**Figure 1:**
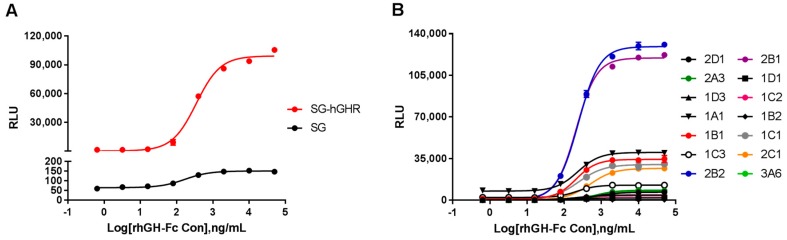
The responsiveness of transfected HEK293 cells to Fc-fusion recombinant human growth hormone (rhGH-Fc). (**A**) The responsiveness of stably transfected HEK293 cell pools to rhGH-Fc. The signal-to-noise ratio (SNR) of HEK293 cells expressing SG-hGHR was significantly higher than that of those having the SG response element. (**B**) The establishment of a responsive HEK293-Luc cell line to rhGH-Fc. Clonal cell line 1B1 was superior to other thirteen clonal cell lines in term of its 50% effective concentration (EC_50_) and SNR. SG-hGHR, SIE and GAS response elements (SG) and human growth hormone receptor (hGHR).

To obtain a stable rhGH-Fc-responsive cell line for the determination of the bioactivity of rhGH-Fc, transfected HEK293 cells expressing SG-luciferase and hGHR (SG-hGHR), and SG-luciferase without hGHR (SG) were constructed. The two cell lines were evaluated for luciferase activity after rhGH-Fc stimulation. The pre-diluted starting concentration of rhGH-Fc was 50,000 ng/mL, and then it was diluted with a dilution ratio of 1:5. Figure 1A showed that sigmoidal curves increased dose-dependently with increasing rhGH-Fc, and the cells expressing SG-hGHR had a better SNR (SNR of 104.0) than control cells that did not express hGHR (SNR of 2.2). In the fourteen clonal cell lines from single cells obtained by limiting dilutions (Figure 1B), a representative cell line, 1B1 (named HEK293-Luc), was chosen for further method validation due to having the highest SNR (SNR of 221.9), a low EC_50_ (207.9 ng/mL), and rhGH-Fc-dependent behavior. 

### 3.2. Method Development and Optimization

**Figure 2:**
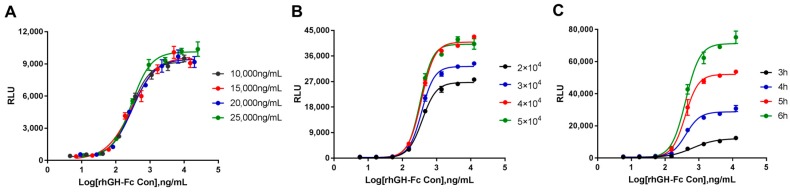
Optimization of experimental parameters of the bioassay. (**A**) Final working concentration of rhGH-Fc. (**B**) Cell numbers per well. (**C**). Incubation time with HEK293-Luc cells and rhGH-Fc.

To optimize the bioassay conditions, three parameters were investigated: the initial concentration of rhGH-Fc, the number of cells per well, and the incubation time. First, the linear region of the dose–response curve of rhGH-Fc was determined by luciferase activity induced by rhGH-Fc and was set at approximately 30.5–15,625 ng/mL (data not shown). The pre-diluted starting concentration of rhGH-Fc was set from 10,000 ng/mL to 25,000 ng/mL with three dilution factors. All of the sigmoidal curves illustrated a dose-dependent trend, and the EC_50_ (276.0–309.5 ng/mL) was similar at the four pre-diluted starting concentrations (Figure 2A). According to the SNR and uniformity of the concentration points, the optimal pre-diluted starting concentration was 15,000 ng/mL (SNR of 119.6). As can be seen from Figure 2B, all of the dose-dependent curves and EC_50_ values were nearly identical (334.4–380.7 ng/mL). The highest magnitude of RLU was illustrated between 4 × 10^4^ and 5 × 10^4^ cells per well, and a higher SNR (SNR of 210.6) was observed with 4 × 10^4^ cells per well. We therefore chose 4 × 10^4^ cells per well as the optimal cell number. Finally, the RLU of the top asymptote increased with an increasing incubation time, and no significant differences were observed in the EC_50_ between 4 and 6 h. Due to its higher SNR (SNR of 107.5), 5 h was deemed to be an applicable incubation time under the given conditions (Figure 2C).

### 3.3. Method Validation

A comprehensive validation of the bioassay was performed according to the ICH Q2 (R1) guidelines and the Chinese Pharmacopoeia [24,25].

#### 3.3.1. Linearity

**Figure 3:**
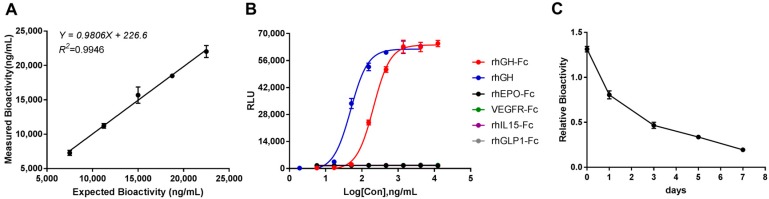
The validation of linearity and specificity. (**A**) A linearity plot of the expected bioactivity against the measured bioactivity. (**B**) The specificity of the bioassay to rhGH-Fc, rhGH, and other therapeutic Fc-fusion proteins. (**C**) The specificity of the bioassay to the thermal degradation of rhGH-Fc. The representative linearity plots of three independent experiments are shown.

The linearity of the bioactivity for rhGH-Fc was evaluated by analyzing the linear curves and CV% using the measured bioactivity at five levels (50%, 75%, 100%, 125% and 150%) of pre-diluted starting working concentration of rhGH-Fc, i.e., 15,000 ng/mL. The measured bioactivities were obtained three times per experiment from three independent experiments applied on three different days and were analyzed using a linear regression model. The linearity plots for the measured bioactivity of rhGH-Fc versus the expected bioactivity displayed a linear behavior (Figure 3A). The CV% of measured bioactivity at five level concentrations ranged from 1.32% to 6.47%, i.e., less than 7.00%. 

#### 3.3.2. Precision

The intra-day and inter-day precision were estimated by analyzing the relative bioactivity of rhGH-Fc samples in triplicate on the same day (intra-day precision) for three different days (inter-day precision). A batch of the final rhGH-Fc product and three freeze-thawed final rhGH-Fc samples were used for this purpose. The precision was expressed by CV% (shown in Table 1). The CV% of intra-day and inter-day precision were lower than 9.00% and 6.00%, respectively, indicating good precision and repeatability of the bioassay. The intermediate precision was determined by a comparison of relative bioactivity performed by two analysts on different days. The relative bioactivity of the seven final rhGH-Fc samples provided a consistent performance, as assessed by the two analysts (the mean relative bioactivity was 1.06 for analyst A, and 1.09 for analyst B). Moreover, the intra-plate of precision based on five repeated analyses for one rhGH-Fc sample in the same 96-well plate was 4.49%. 

#### 3.3.3. Accuracy

The accuracy of the described method was evaluated by the definite addition of an in-house reference in a sample, and it is indicated in terms of recovery (%) [20]. Here, the recovery was calculated using the rhGH-Fc spiking method at 50% of the level of the in-house reference concentration. The assay was performed three times per day on three different days. The mean recovery of the in-house reference was 113.64%, and the 95% confidence interval (CI) of that was 106.00–121.30%. The maximum CV% of the intra-day precision was 7.79%, and that of the inter-day precision was 8.73% (Table 2).

#### 3.3.4. Specificity

The specificity of the method was confirmed by the responsiveness of HEK293-Luc cells to other Fc fusion therapeutic drugs, e.g., rhEPO-Fc, VEGFR-Fc, rhIL15-Fc, and rhGLP1-Fc (Figure 3B). None of them had any effect on HEK293-Luc cells. On the other hand, an expected result for rhGH with a standard sigmoidal curve parallel to rhGH-Fc was observed. Further, we forced the degradation of rhGH-Fc by thermal stress [20,24,26]. After incubation at 60 °C for different number of days, we saw a gradual decline in the relative bioactivity of rhGH-Fc as the number of days increased, which ranged from 1.31 in the normal sample to 0.19 in the degraded rhGH-Fc sample at 7 days (Figure 3C).

#### 3.3.5. Robustness

**Figure 4:**
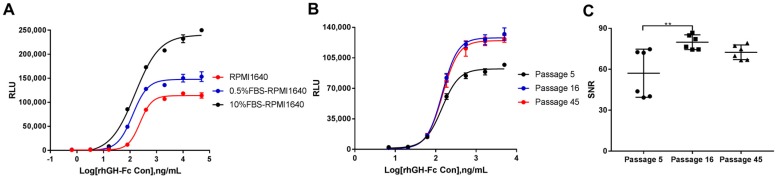
The robustness of the responsive HEK293-Luc cell line to rhGH-Fc. (**A**) The responsiveness of HEK293-Luc cells to rhGH-Fc in 0, 0.5%, and 10% FBS RPMI 1640 assay media. (**B**) The sigmoidal curves of HEK293-Luc cells responded to rhGH-Fc at passage 5, passage 16, and passage 45. (**C**) The SNRs of HEK293-Luc cells responded to rhGH-Fc at passage 5, passage 16, and passage 45. ** *p* < 0.01.

To investigate the robustness of the bioassay, some changes to the assay media were made. As shown in Figure 4A, the responsiveness of HEK293-Luc cells to rhGH-Fc in FBS-free RPMI 1640 was observed. The RLU values of top asymptotes in assay media supplemented with 10%, 0.5%, and 0% FBS decreased gradually. No significant changes in the SNR were found because of the different RLU values of the bottom asymptotes (the mean RLUs were 1425.4, 896.8, and 675.7, respectively). Additionally, the results of the stability of HEK293-Luc cells are shown in Figure 4B,C. Parallel dose–response sigmoidal curves were found between passage 5 and passage 45 (Figure 4B). The EC_50_ values of passage 5, passage 16, and passage 45 were similar: 141.1, 145.3, and 147.9 ng/mL, respectively. The SNR of passage 16 was significantly higher than that of passage 5 (*p* = 0.0087), having changed from 57.13 to 79.90. As expected, the SNR of passage 45 (72.48) was consistent with that of passage 16 (Figure 4C).

## 4. Discussion

It is noteworthy that several rhGH-Fc types are at different stages of clinical and preclinical development [7,16]. The bioactivity of rhGH-Fc plays a role in quality control and is required to establish a national or international bioassay. In the published literature, a few studies focused on the proliferation assay and the hGHR-LHRE reporter system in some cell lines, e.g., HEK293, Ba/F3, and CHO, but the analytical method has not previously been fully validated. Moreover, transient transfection is not suitable for determination the bioactivity of therapeutic rhGH or LAGH [18,21,22,23,27]. Here, we developed and validated an animal-free bioassay based on RGA for the determination of the bioactivity of rhGH-Fc. It is the first time that the validity of this bioassay to determine the bioactivity of rhGH-Fc has been stringently validated. 

The RGAs consist of a reporter gene controlled by a specific regulatory response element and receptors expressed on the cell surface. In this study, the reporter gene system was indicated by firefly luciferase and the SG response element associated with the JAK2-STATs signaling pathway [4,19,28,29]. The binding of GH to its receptor can activate JAK2, stimulate tyrosyl phosphorylation of both GHR and JAK2, and initiate signaling pathways. The STATs pathway is important to allow GH to exert physiological effects. Phosphorylated STAT5, a major molecule in the STATs signaling pathway, translocates to the nucleus, where it binds to the SG response element promoter, including SIE and GAS, activates the expression of the downstream gene, and induces cell differentiation and proliferation [2,3,4,30,31,32,33,34]. 

During the development phase, the parameters listed in Section 3.2 were optimized based on the SNR and EC_50_ using traditional “single factor at a time” experiments. Then, a comprehensive validation method was carried out to assess the validity of the bioassay according to the ICH Q2 (R1) guidelines and Chinese Pharmacopoeia. Specificity is considered to be the most important parameter, and it was assessed using other Fc fusion proteins and degraded compounds of rhGH-Fc. The rhGH-Fc and rhGH appeared to exhibit remarkable specificity for hGHR and SG. Likewise, the bioassay showed high specificity in discriminating the bioactivity of degraded rhGH-Fc. 

For the validation of cell-based bioassay, there are no acceptance criteria for precision and accuracy in the ICH Q2 (R1) guidelines. Hence, we used CV of 15% to 20% as the acceptance criteria, as recommended by the AAPS/FDA Bioanalytical Workshop [35]. In our study, all of the %CV values (intra-day and inter-day), including the precision, accuracy, and linearity, were less than 9.00%, which suggests that this method is acceptable for the bioactivity determination of rhGH-Fc. Moreover, the bioactivity determined by two analysts suggested that the bioassay was characterized by excellent intermediate precision. As expected, the bioassay was unaffected by some deliberate changes in method parameters. For one thing, parallel EC_50_ and SNR responses of HEK293-Luc to rhGH-Fc were observed in the two-assay media, i.e., the DMEM supplemented with 20 mM HEPES and the commercial RPMI 1640 with HEPES (data not shown). Due to its easy-to–use nature, we chose the RPMI 1640 without additional HEPES as the assay media. Additionally, the FBS-free RPMI 1640 media with an identical SNR and decreasing background luciferase values was optimal. The responsiveness of the HEK292-Luc cell line to rhGH-Fc was considered stable between passage 16 and passage 45. It is noteworthy that there is still the risk of potential contaminants of SG inhibitors or activators in the samples or media, although FBS was excluded from the assay media during the bioassay procedure. Generally, any contaminant in media only affects the background signals, but has little influence on the dose–response curve. As for the samples, which are always final products, their purities and impurities should be qualified to reduce the risk of contaminants. 

Taken together, a simple and time-saving bioassay was developed for the determination of rhGH-Fc bioactivity. The proposed bioassay was proven to be precise, reproducible, and robust. It has the potential to be used for the routine analysis of bioactivity during the research, development, and manufacture of therapeutic rhGH-Fc, probably for other LAGH products. Further, the bioassay could probably be used as an alternative bioassay to the traditional in vivo rat weight gain assays; however, an agreement study of the two methods to determining the GH bioactivity is required.

## Figures and Tables

**Table 1:** The precision of the rhGH-Fc bioactivity results.

	Intra-Day CV%			Inter-Day CV%	95% CI *^1^ of Mean Relative Bioactivity
	1	2	3		
rhGH-Fc	2.42	3.82	5.00	2.06	1.10–1.17
rhGH-Fc D1 *^2^	2.71	4.16	5.96	4.74	1.00–1.09
rhGH-Fc D3 *^2^	2.04	5.05	8.81	2.27	0.80–0.87
rhGH-Fc D5 *^2^	7.60	3.01	2.16	5.87	0.93–1.03

*^1^ CI: confidence interval. *^2^ The final rhGH-Fc product was frozen at –80 °C and thawed at 25 °C for 1, 3, and 5 cycles, respectively.

**Table 2:** The results of recovery for an in-house rhGH-Fc reference.

	Recovery (%)			Intra-day CV%	Mean	SD *
	1	2	3			
1	127.21	130.54	112.49	7.79	123.41	9.61
2	112.69	115.19	101.98	6.38	109.95	7.02
3	100.96	111.20	110.52	5.32	107.56	5.73
Mean	113.64					
SD *	9.92					
Inter-day CV%	8.73					

* SD: Standard deviation.
